# Cross-sectional study of the relationship of peripheral blood cell profiles with severity of infection by adenovirus type 55

**DOI:** 10.1186/1471-2334-14-147

**Published:** 2014-03-19

**Authors:** Wei-Wei Chen, Wei-Min Nie, Wen Xu, Yang-Xin Xie, Bo Tu, Peng Zhao, En-Qiang Qin, Yun-Hui Zhang, Xiu Zhang, Wen-Gang Li, Zhi-Ping Zhou, Ji-Yun Lv, Min Zhao

**Affiliations:** 1 School of Management, University of Chinese Academy of Sciences, No. 80 East Road Zhongguancun, 100190 Beijing, China; 2 Treatment and Research Center for Infectious Diseases, 302 Military Hospital of China, No. 100 West 4th Ring Middle Road, 100039 Beijing, China

**Keywords:** Infectious diseases, Adenovirus, Immunopathology, Outbreak

## Abstract

**Background:**

The immunologic profiles of patients with human adenovirus serotype 55 (HAdV-55) infections were characterized in subjects diagnosed with silent infections (n = 30), minor infections (n = 27), severe infections (n = 34), and healthy controls (n = 30) during a recent outbreak among Chinese military trainees.

**Methods:**

Blood was sampled at the disease peak and four weeks later, and samples were analyzed to measure changes in leukocyte and platelet profiles in patients with different severities of disease. Differential lymphocyte subsets and cytokine profiles were measured by flow cytometry and Luminex xMAP®, and serum antibodies were analyzed by ELISA and immunofluorescence staining.

**Results:**

Patients with severe HAdV infections had higher proportions of neutrophils and reduced levels of lymphocytes (*p* < 0.005 for both). Patients with minor and severe infections had significantly lower platelet counts (*p* < 0.005 for both) than those with silent infections. The silent and minor infection groups had higher levels of dendritic cells than the severe infection group. Relative to patients with silent infections, patients with severe infections had significantly higher levels of IL-17^+^CD4^+^ cells, decreased levels of IL-17^+^CD8^+^ cells, and higher levels of IFN-γ, IL-4, IL-10, and IFN-α2 (*p* < 0.001 for all comparisons).

**Conclusions:**

Patients with different severities of disease due to HAdV-55 infection had significantly different immune responses. These data provide an initial step toward the identification of patients at risk for more severe disease and the development of treatments against HAdV-55 infection.

## Background

Febrile respiratory illness (FRI) due to human adenovirus (HAdV) infection has become a leading cause of morbidity in military trainees since the cessation of HAdV vaccine production in 1999 [1-4]. There are 65 known serotypes of HAdV and different serotypes are associated with pneumonia, severe respiratory failure, gastrointestinal disorders, or ocular diseases. One study of an FRI outbreak in U.S. Air Force trainees reported that almost half of the subjects were infected with HAdV-14, some required hospitalization, and one person died [5]. Another study reported low morbidity and mortality of U.S. trainees following an outbreak of HAdV-14 pneumonia, but a higher hospitalization rate for females [4]. Given the rapid spread and high infectivity of HAdV and the mobility and close contact of military trainees, it is crucial to identify biomarkers with diagnostic or prognostic value.

Genome-wide computational analysis indicated that HAdV-55, originally termed an “HAdV-B11-like” virus [6,7], arose as a new pathogen due to recombination of HAdV-11 and HAdV-14 [6,8]. From 2004–2006, outbreaks occurred in Turkey, Singapore, and China (incorrectly diagnosed as HAdV-11 at that time), with two of these in military training bases [9,10] and one in a school [7]. It is unclear why HAdV-55 infection only leads to severe pneumonia in certain patients, and there are currently there no effective treatments.

In humans and *in vivo* models, the progression of AdV infection is associated with changes in inflammatory cells, and the inflammatory infiltrate is initially composed of neutrophils and then monocytes [11,12]. As disease progresses, lymphocytic inflammation occurs, a response due to the innate and adaptive immune systems. The accompanying release of proinflammatory cytokines, including TNF-α, IL-6, IL-1β, and IFN-γ [11,13], is likely responsible for the resulting tissue injury in some cases [12]. However, changes in inflammatory cell counts may be serotype-specific, because some patients with HAdV-14 infections have significantly reduced counts of white blood cells, neutrophils, lymphocytes, and platelets relative to those without infection [4]. Certain AdV serotypes can suppress the immune response [14,15]. For example, HAdV-35 suppresses CD4^+^ T cell activation through suppression of CD46 expression [14], and HAdV-5 suppresses IFN production through posttranslational modification [15]. To date, there has been no analysis of the immunological response to HAdV-55 infection.

DNA sequencing is useful for HAdV identification, but viral sequence alone does not determine the risk for development of severe disease. The objective of the present study was to examine Chinese military trainees from one of the aforementioned HAdV outbreaks [16] and to identify clinical and laboratory markers that have potential diagnostic or prognostic value. This is the first study to analyze the peripheral blood cell profiles of patients with varying severities of HAdV-55 infection at the onset of an outbreak and four weeks after onset. The results will provide insight into the pathogenesis of severe disease due to HAdV-55 infection and help to identify markers that may ultimately be used for early diagnosis and treatment of HAdV-55 infection.

## Methods

### Study population and diagnostic criteria

Two continuous outbreaks occurred at two military camps (December 2011 to January 2012, February 2012 to March 2012), during which about 1000 soldiers were diagnosed with a HAdV-55 infections. This study analyzed 30 patients with silent infections, 27 with minor infections, 34 with severe infections, and 30 healthy control subjects by convenience sampling. The healthy control subjects were recruited from a different military base in which there was no disease outbreak. All subjects were males and 17 to 27 years-old (Table 1). Samples were collected by throat swabs, and HAdV-55-specific DNA was detected by real-time quantitative PCR as described below. One blood sample was taken from subjects in the healthy control and minor infection groups at the peak of the outbreak (the acute phase [AP]); blood samples were taken at the AP and four weeks later (the convalescent phase [CP]) in the silent and severe infection groups. The Ethics Committee of 302 Military Hospital of China approved this study and all participants provided informed written consent.

**Table 1:** Characteristics of patients diagnosed with adenovirus type 55 infections

	**Healthy control (n = 30)**	**Silent infection (n = 30)**	**Minor infection (n = 27)**	**Severe infection (n = 34)**
**Age range, years**	18-25	17-21	17-24	17-27
**HAdV-spec IgG**^**+**^**, % (n)**	0 (0/30)	100 (30/30)	100 (27/27)	100 (34/34)
**HAdV55-spec IgM**^**+**^**, % (n)**	0 (0/30)	90.0 (18/20)	86.7 (13/15)	100 (34/34)
**HAdV-55 DNA**^**+ **^**% (n)**	0 (0/30)	3.3 (1/30)	30 (9/30)	41.2 (14/34)

Clinical and laboratory parameters were collected from all patients and used for diagnosis [17]. A minor infection was defined by one of the following conditions: upper respiratory tract infection; fever with dry throat or sore throat; dry cough and throat congestion; lymphofollicular hyperplasia; spotty or flaky off-white secretions on the tonsil surface; normal or declined white blood cell count in the peripheral blood; decreased proportion of lymphocytes; or increased proportion of monocytes. A severe infection was defined by the same conditions in addition to nodular, patchy, or large areas of consolidations in lung imaging. Silent infection was defined as the absence of clinical symptoms, but positive results for AdV-specific IgM, as described below.

### Pathogen isolation and serotype identification

Thirty throat swabs were collected from patients in the severe infection group and were used to inoculate human adenocarcinoma A549 cells. In the first inoculation, 10 A549 cultures were inoculated with samples from 30 patients (three patients per culture dish). After the cells were passaged twice, typical cytopathic effects (CPEs), including cell rounding and grape-like clustering with enhanced light refraction, occurred in five culture dishes (samples from 15 patients). In the second inoculation, samples from these 15 patients were individually inoculated into A459 cells. Positive viral isolates were defined by the appearance of CPEs and positive nucleic acid tests. Cells were harvested for nucleic acid measurements when CPEs were observed in 76-100% of cells. If CPEs were absent after cells were cultured for three generations, and no viral nucleic acid was detected, the isolate was considered negative. A total of eight viral clones were ultimately isolated.

For inoculation, samples were placed in tubes containing 2 mL of HBSS (Sigma-Aldrich, St Louis, MO, USA), and these tubes were oscillated for 30 sec and then centrifuged at 1500 rpm for 10 min. The supernatant was collected, 1000 U/mL each of penicillin and streptomycin (Life technologies, Carlsbad, CA, USA) were added, the sample was incubated at 4°C for 2 h, and the solution was inoculated onto a monolayer of A549 cells. The cells were cultured in DMEM that contained 10% fetal bovine serum and 100 U/mL each of penicillin, streptomycin, and kanamycin (all from Life Technologies) at 32°C with 5% CO_2_, and examined by microscopy daily.

Nucleic acids were extracted using the QIAmpMiniElute Virus Span kit (Qiagen, Hilden, Germany), following manufacturer’s instructions. Three sets of PCR primers were used to confirm that the isolated virus was an adenovirus: an AdV general primer pair [18], a primer pair for the AdV B group Hexon, and a primer pair for the AdV B group Fiber [19] (Table 2). Purified viral genomic DNA was used as the template for the PCR reaction that included the following reagents in 25 μL: 10 × buffer (2.5 μL), 10 mM dNTP mix (2 μL), 50 mM upstream and downstream primers (0.5 μL each), DNA polymerase (0.3 μL), and the DNA template (2 μL). The thermocycling regimen was: one cycle at 94°C for 3 min, 35 cycles at 94°C for 30 sec, 55°C for 45 sec, 72°C for 45 sec, and a final elongation step at 72°C for 10 min. The amplification products were separated by 2% agarose gel electrophoresis. The sequence of HAdV-55 is 98.86% identical to HAdV-14 [6,8], so HAdV-55 identification was confirmed by sequencing of PCR products (Life Technologies) to identify the virus serotype and the sequence was submitted to the NCBI database for BLAST analysis and to the gene bank of the Chinese PLA Center for Disease Control and Prevention. Table 1 shows the proportion of samples that were positive for HAdV-55 nucleic acids.

**Table 2:** PCR primers used in RT-PCR analysis

**Target**	**Direction**	**Sequence (5′→3′)**	**Product size (bp)**
Universal	Forward	TTCCCCATGGCICAYAACAC	482
	Reverse	CCCTGGTAKCCRATRTTGTA	
Hexon	Forward	TTGACTTGCAGGACAGAAA	590
	Reverse	CTTGTATGTGGAAAGGCAC	
Fiber	Forward	TACCCCTATGAAGATGAAAGCA	1064
	Reverse	GGAGGCAAAATAACTACTCG	

### Detection of serum antibodies

HAdV55 is a new strain of virus, so no HAdV-55-specific IgG ELISA kit is available. Thus, we used a commercially available HAdV-specific IgG ELISA kit (RE56571 Adenovirus IgG; IBL, Hamburg, Germany) to measure the increase of HAdV-specific IgG during the acute and convalescent phases An AdV-specific IgG level greater than 12 U/mL is defined as positive according to the manufacturer’s instruction. Subsequently, all cases with 4-fold increase in AdV-specific IgG were further confirmed by HAdV-55-spec IgG immunofluorescence as the methods described below. Another adenovirus ELISA kit (RE56581 Adenovirus IgM; IBL) is specific for adenovirus type 5 Hexon antigen, but not for HAdV-55, so detection of HAdV-55-specific IgM (HAdV55-IgM) was performed by an immunofluorescence (IF) assay. A549 cells were inoculated with the isolated virus strains to prepare viral antigen slides for detection of serum IgM and IgG by IF assay. Specifically, A549 cells with positive viral isolation results were freeze-thawed, and a 1 × 10^−3^ dilution was used to inoculate the A549 cells. When CPEs occurred in ~50% of cells, they were harvested, washed with PBS (pH 7.4) three times, and fixed with cold acetone overnight. After drying, the antigen slides were divided into small pieces (0.3 × 0.3 cm^2^) and stored at -20°C until use. Uninfected A549 cell slides were prepared as controls.

For IF analysis, antigen slides were fixed on a glass slide with the cell side facing up, washed three times with PBS, blocked with bovine serum albumin for 20 min, and then incubated with 50 μL of diluted patient serum (1:20 for IgM and 1:50 ~ 1: 800 for IgG in PBS). Slides incubated with 50 μL PBS served as a negative control. Samples were incubated in a moist chamber at 4°C overnight, washed with three times with PBS, and incubated with FITC-conjugated rabbit anti-human IgM (ZF-0307, 1:150 dilution; OriGene, Rockville, MD, USA) or anti-human IgG (ZF-0308, 1:150 dilution; OriGene). After incubation in a moist chamber at 37°C for 40 min and a rinse with PBS, nuclei were stained with Evans blue, and slides were mounted with glycerol and observed by fluorescence microscopy (Nikon-80i, Japan) at 400× magnification. Table 1 shows the proportion of patients who were positive for adenovirus specific HAdV-IgG and HAdV55-IgM. The HAdV-55-IgM fluorescence intensity was scored from 0 (no positive cells) to 4 (highest fluorescence). Each slide was scored by two experienced pathologists, and average scores were recorded.

### Analysis of blood cells and lymphocyte subsets

Hematologic tests were performed using Sysmex XE 2100 (Kobe, Japan). Flow cytometry used fluorophore-conjugated antibodies (BD Biosciences, San Jose, CA, USA) and FACSCalibur and CELLQuest software (BD Biosciences) to detect lymphocyte subsets, dendritic cells (DCs), IL-17^+^ T cells, and activated T cells. Peripheral blood lymphocyte (PBL) subsets were determined using a MultiTEST IMK kit (BD Biosciences) following the manufacturer’s instructions. CD3^+^ T lymphocytes were CD3^+^CD45^+^; CD4^+^ T lymphocytes were CD3^+^CD4^+^CD45^+^; CD8^+^ T lymphocytes were CD3^+^CD8^+^CD45^+^; B lymphocytes were CD3^−^CD19^+^ CD45^+^; and natural killer (NK) cells were CD3^−^CD16^+^CD56^+^CD45^+^. DCs were identified using a combination of CD123-PE, HLA-DR-Percp, Lin-1-FITC, and CD11c-APC antibodies. Plasmacytoid DCs (pDCs) were Lin-1^−^HLA-DR^+^CD123^+^ and myeloid DCs (mDCs) were Lin-1^−^HLA-DR^+^CD11c^+^. A combination of antibodies specific for CD3-APC, CD8-Percp, IFN-γ-FITC, and IL-17-PE were used to identify Th17 cells. Activated T cells were identified using a combination of CD3-APC, CD8-Percp, CD38-FITC, and CD56-PE antibodies. Data were analyzed with FlowJo 7.0 software.

### Analysis of cytokine levels

Cytokine levels were assessed using the FLEXMAP 3D system (Luminex, Austin, TX, USA) and a human cytokine kit (Milliplex, Catalog no. MPXHCYTO-60 K-16, Millipore, Billerica, MA, USA) following the manufacturer’s instructions. The selected cytokines were Fractalkine, IFN-α2, IFN-γ, IL-1β, IL-2, IL- 4, IL-5, IL-6, IL-8, IL-10, IL-15, IL-17, MCP-3., MIP1-A, MIP1-B, TNF-α, and IL-12.

### Statistical analysis

**Figure 1:**
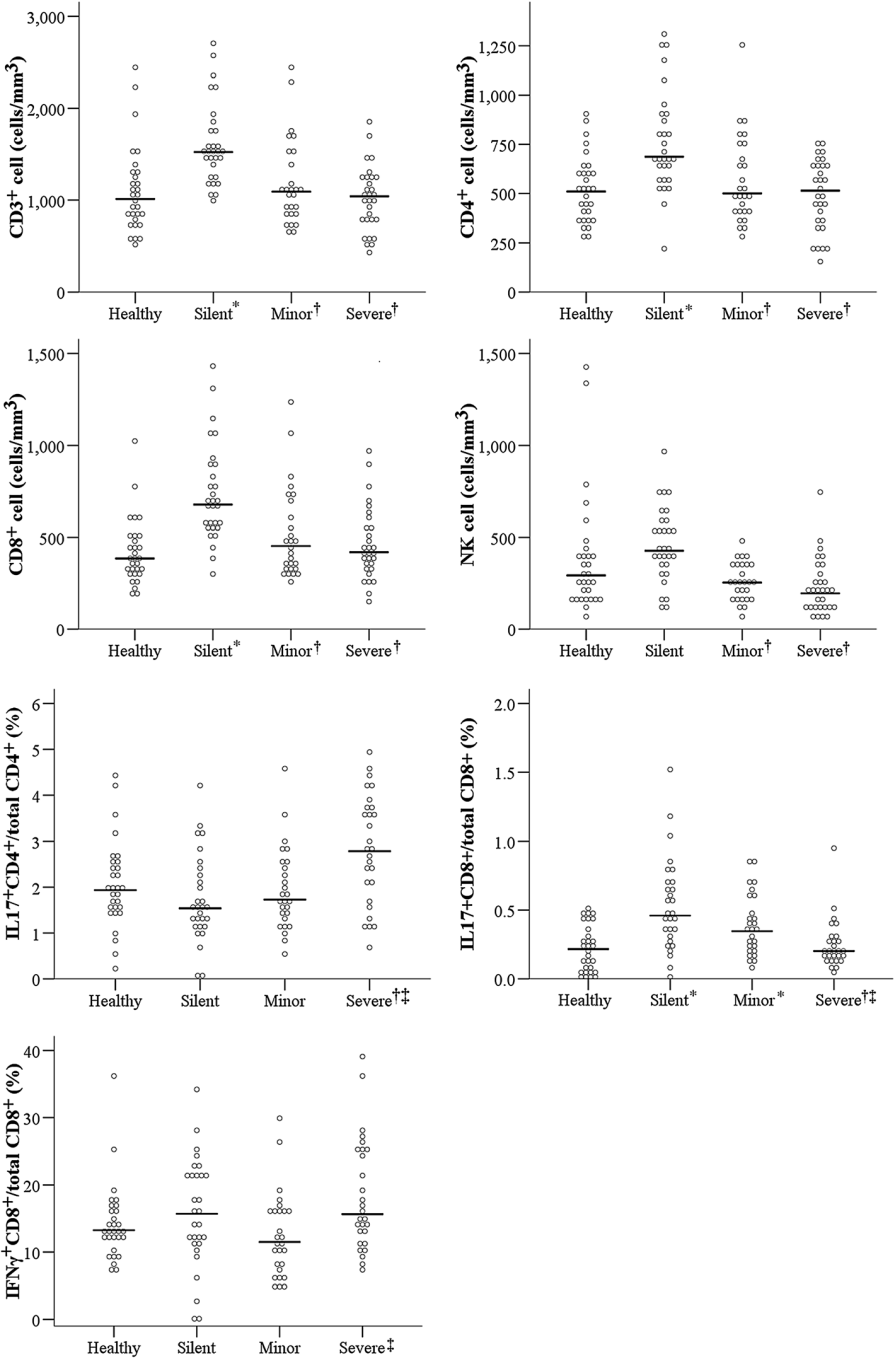
**Lymphocyte subsets of the different groups of patients in the AP phase, determined using flow cytometry.** Here and below, solid horizontal lines indicate medians. ^*^Significantly different from the healthy control group. ^†^Significantly different from the silent infection group. ^‡^Significantly different from the minor infection group.

**Figure 2:**
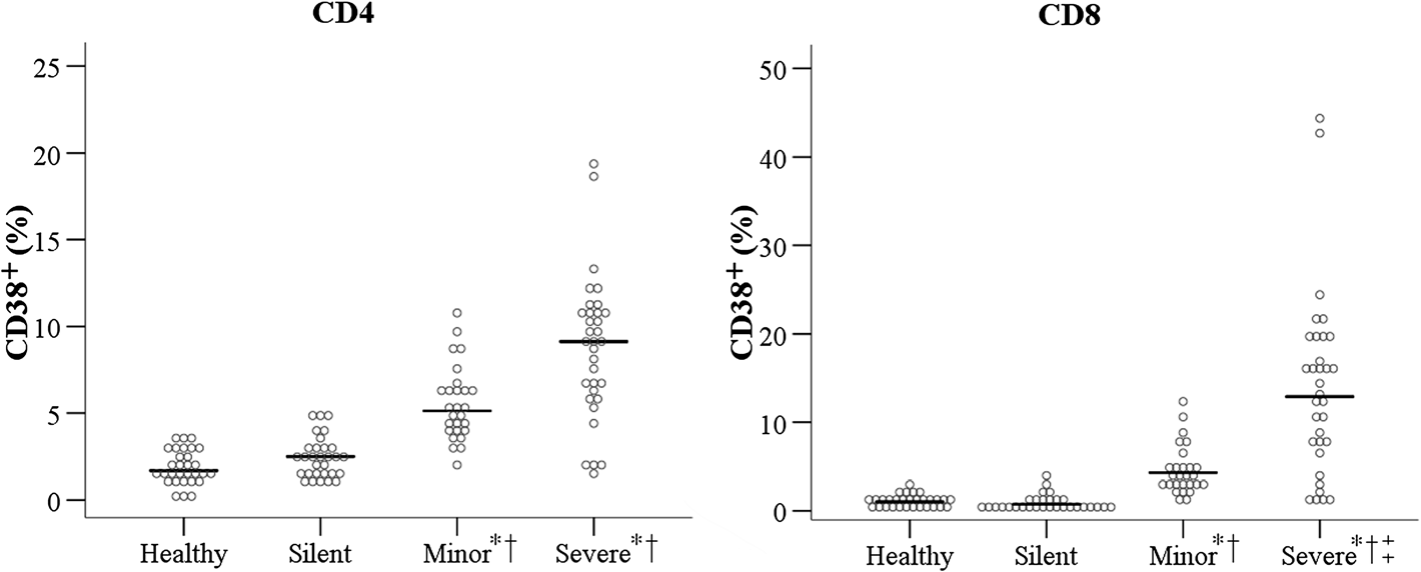
**Percentage of CD38**^**+ **^cells of the different groups of patients in the AP phase. ^*^Significantly different from the healthy control group. ^†^Significantly different from the silent infection group. ^‡^Significantly different from the minor infection group.

**Figure 3:**
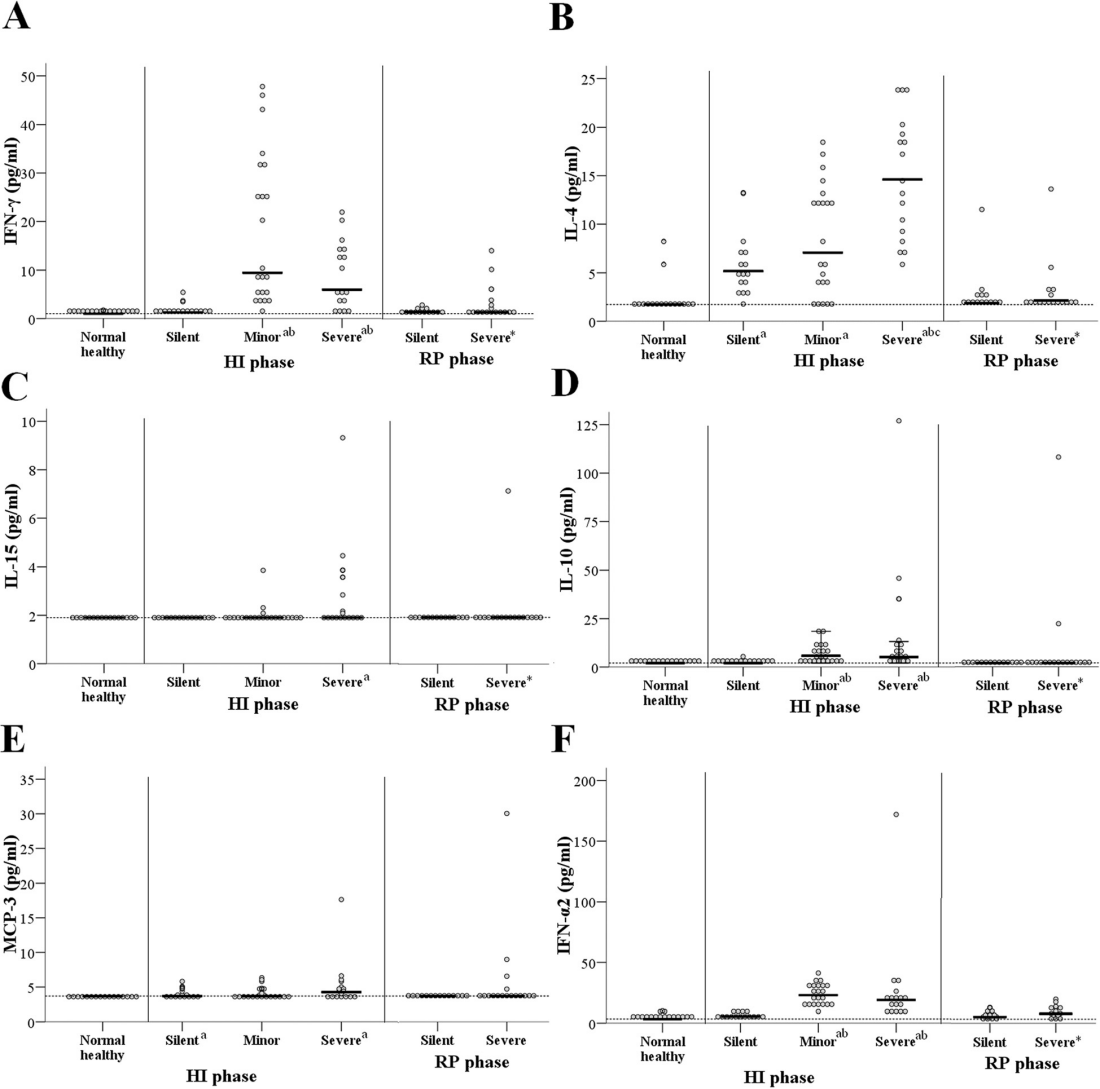
**Cytokine levels in the AP and CP phases of the different groups of patients. (A)** IFN-γ, **(B)** IL-4, **(C)** IL-15, **(D)** IL-10, **(E)** MCP-3, and **(F)** IFN-α2. The dotted line indicates the lower limit of detection. ^a^Significantly different from the control group. ^b^Significantly different from the silent infection group at the AP phase. ^*^Significantly different from the baseline level within group (CP phase vs. AP phase).

Categorical data are presented as counts and percentages and continuous data as means and standard deviations (SDs). Comparisons among groups were performed using one-way ANOVA with the Bonferroni *post hoc* correction. Due to the small sample size, Figures 1, 2 and 3, and Additional file 1: Tables S2 and Additional file 1: S3 show the baseline lymphocyte and CD38 levels and cytokine levels at two phases with medians and range. Comparisons among groups were performed by the non-parametric Kruskal-Wallis test and when the results were statistically significant, multiple tests for pairwise comparisons with the Bonferroni correction were performed using the non-parametric Mann–Whitney test. The significance of within-group differences of the two disease phases (AP *vs.* CP) were assessed using the Wilcoxon signed-rank test. A two-tailed *p*-value less than 0.05 was considered statistically significant. Statistical analyses were performed with SPSS 15.0 (Chicago, IL, USA).

## Results

### Diagnosis of HAdV-55 infection

We analyzed patients for the presence of HAdV-55 infection based on antibody titer and the presence of viral DNA (Table 1). All samples from the control group were negative in all 3 tests. HAdV specific IgG was present in 100% of the samples of the three infection groups. HAdV55-specific IgM was present in 90% of patients with silent infections, 86.7% of patients with minor infections, and 100% of patients with severe infections. HAdV-55 DNA was present in 3.3% of patients with silent infections, 30% of patients with minor infections, and 41.2% of patients with severe infections.

### Effect of HAdV-55 infection on blood cell counts

The four groups had similar WBC counts during the AP, but there were significant differences in the proportions of neutrophils, lymphocytes, monocytes, and platelets during this phase (Table 3). In particular, the severe infection group had a greater proportion of neutrophils than the silent infection and minor infection groups (*p* < 0.005 for both), and a smaller proportion of lymphocytes than the silent infection group (*p* < 0.005). The minor and severe infection groups had significantly higher proportions of monocytes than the control and silent infection groups (*p* < 0.005 for all). The silent infection group had significantly higher PLT counts than the healthy group, the minor infection group, and the severe infection group (*p* < 0.005 for all).

**Table 3:** Summary of blood cell types in the different groups at the acute phase (AP) and receding pandemic (CP) phases

	**Healthy controls (n = 30)**	**Phase**	**Silent infection (n = 30)**	**Minor infection (n = 27)**	**Severe infection (n = 34)**	***P*-value**
**WBC, 10**^**9**^**/L**	5.18 ± 1.21	AP	6.03 ± 1.13	5.49 ± 1.58	6.21 ± 2.96	0.1333
		CP	6.17 ± 1.32^b^	ND	5.78 ± 1.40	0.0164^a^
**Neutrophil, fraction**	0.57 ± 0.08	AP	0.51 ± 0.09	0.51 ± 0.14	0.61 ± 0.15^c,d^	0.0020^a^
		CP	0.52 ± 0.08	ND	0.50 ± 0.09^b,e^	0.0026^a^
**Lymphocyte, fraction**	0.34 ± 0.07	AP	0.38 ± 0.08	0.34 ± 0.15	0.27 ± 0.13^c^	0.0009^a^
		CP	0.36 ± 0.08	ND	0.39 ± 0.08^e^	0.0609
**Monocyte, fraction**	0.07 ± 0.02	AP	0.09 ± 0.02	0.13 ± 0.03^b,c^	0.11 ± 0.04^b,c^	<0.0001^a^
		CP	0.09 ± 0.02	ND	0.09 ± 0.02^e^	0.0001^a^
**PLT, 10**^**9**^**/L**	207.13 ± 36.88	AP	260.40 ± 54.22^b^	213.81 ± 47.54^c^	181.47 ± 48.33^c^	<0.0001^a^
		CP	224.37 ± 39.78^e^	ND	253.00 ± 51.70^b,c,e^	0.0004^a^

AP, acute phase; CP, convalescent phase; ND, not determined; WBC, white blood cells; PLT, platelet.

^a^significant differences in the four groups by one-way ANOVA.

^b^significant difference from the healthy control group.

^c^significant difference from the silent infection group.

^d^significant difference from the minor infection group.

^e^significant difference from the AP phase within a group.

The blood cell counts were very different during the CP (Table 3). Relative to the AP (baseline), the severe infection group had significantly lower proportions of neutrophils (0.61 *vs.* 0.50; *p* < 0.0001) and monocytes (0.11 *vs.* 0.09 and; *p* < 0.05), and a higher proportion of lymphocytes (0.27 *vs.* 0.39, *p* < 0.0001). The severe infection group had greater PLT counts at the CP relative to baseline (253.0 *vs.* 181.47 × 10^9^/L; *p* < 0.0001), but the silent infection group had significantly lower PLT counts relative to baseline (224.37 *vs.* 260.40 10^9^/L, *p* < 0.0001).

### Effect of infection severity on IgM IF score

**Figure 4:**
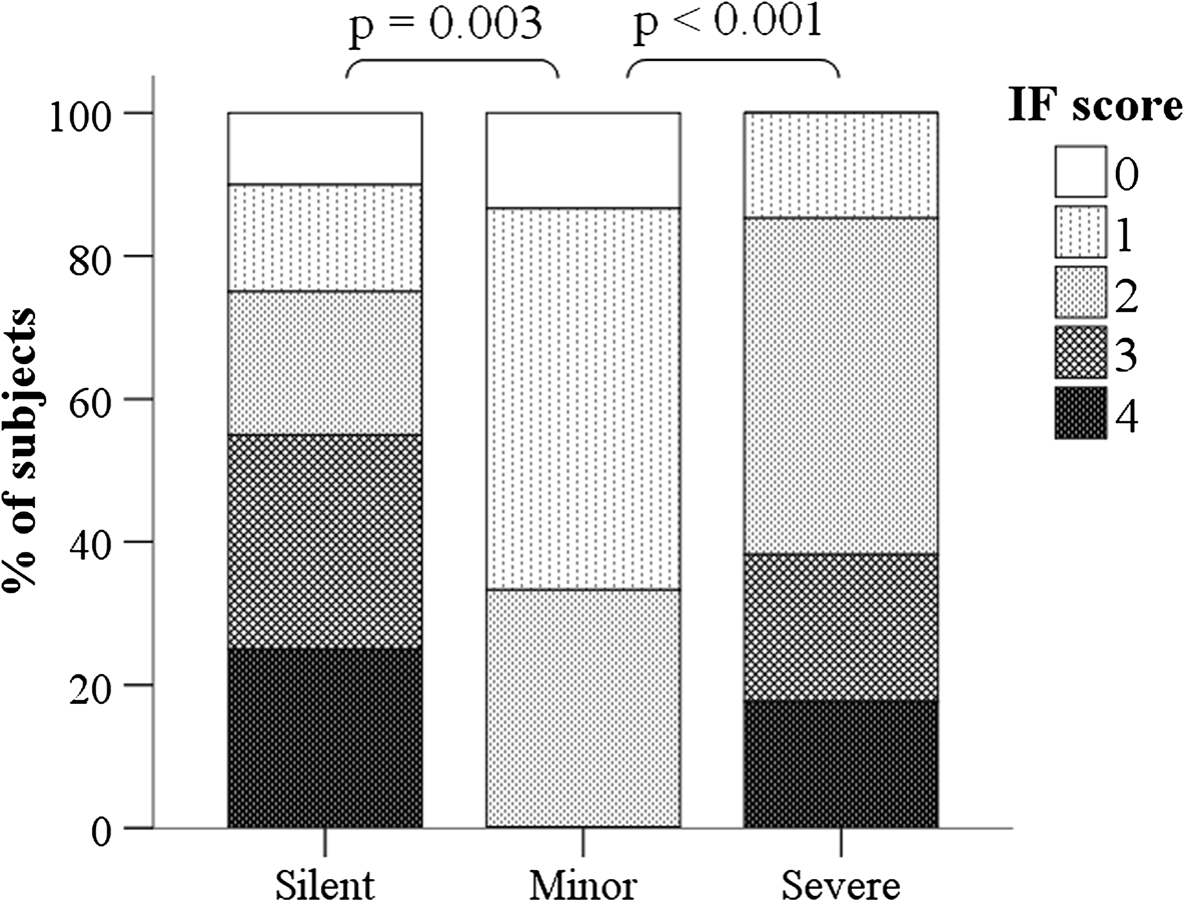
HAdV55-IgM IF scores in subjects with silent, minor, and severe infections.

We also compared the HAdV55-IgM IF scores of the silent, minor, and severe infection groups (Figure 4). As expected, all patients in the control group had IF scores of 0 (data not shown). Somewhat unexpectedly, patients in silent and severe infection groups had significantly higher IF scores than patients in the minor infection group (*p <* 0.003 and *p* < 0.001, respectively). In addition, 55.0% of the silent infection group and 38.2% of the severe infection groups had IF scores of 3 or more, but all patients in the minor infection group had IF scores less than 3. Additional file 1: Table S1 summarizes the changes in Adv-specific IgG titer from the AP to the CP in the patients of silent infection and severe infection groups.

### Lymphocyte subsets in patients and controls

We also analyzed the lymphocyte subsets in all four groups at the AP (Figure 1). Subjects with silent infections had significantly higher numbers of CD3^+^ cells (median: 1523.0/mm^3^), CD4^+^ cells (median: 687.0/mm^3^), and CD8^+^ cells (median: 677.5/mm^3^). There were no significant differences in these three lymphocyte subsets among the other 3 groups. The silent infection group had significantly higher NK cell counts (median: 427.0/mm^3^) than the minor and severe infection groups. The percentage of IFN-γ^+^CD8^+^ cells among all CD8^+^ T cells was significantly higher in the severe infection group than in the minor infection group (median: 15.7 *vs.* 11.5, *p* = 0.006). Additional file 1: Table S2 summarizes the changes in the lymphocyte subsets of each group from the AP to the CP.

We also determined the percentage of activated T cells (CD38+ cells) among peripheral blood lymphocytes (PBL) at baseline (Figure 2). There were more CD38^+^ cells in the minor and severe infection groups than in the healthy and silent infection groups among CD4^+^ cells, but this difference was not statistically significant. There were significantly more CD38^+^ cells in the minor and severe infection groups than in the healthy and silent infection groups among CD8^+^ cells (CD4^+^: 5.13 and 9.14 *vs.* 1.67 and 2.49, respectively, *p* < 0.001; CD8^+^: 4.28 and 12.90 *vs.* 1.03 and 0.75, respectively, *p* < 0.001; Figure 2).

At the CP, the silent and severe infection groups had no significant changes in the counts of CD3^+^, CD4^+^, and CD8^+^ cells relative to baseline; however, these counts were greater than the control group at baseline (*p* ≤ 0.001 for all comparisons; Additional file 1: Table S2). The silent infection group had significantly lower lymphocyte and NK cell counts in the CP than at baseline; however, the severe infection group had significant increases in these counts relative to baseline (*p* < 0.0021). Although the silent infection group had decreased lymphocyte counts at the CP, they remained higher than the healthy control and minor infection groups (means of 2162.40/mm^3^, 1717.93/mm^3^, and 1692.26/mm^3^ respectively). The silent and severe infection groups had significantly higher B cell counts at the CP than at baseline (*p* < 0.05; Additional file 1: Table S2).

### Effects of HAdV-55 infection severity on DC and Th17 cells

Analysis of PBLs indicated that the silent and minor infection groups had significantly higher mDC and pDC levels than the control and severe infection groups in the AP (*p* < 0.05 for all comparisons; Table 4). The severe infection group had significantly higher mDC/PBMC and pDC/PBMC ratios in the CP relative to baseline (0.70 *vs.* 0.53 and 0.38 *vs.* 0.22, respectively).

**Table 4:** Analysis of lymphocyte subsets in the different groups at the acute phase (AP) and receding pandemic (CP) phases

**Lymphocyte subsets**	**Healthy controls (n = 30)**	**Phase**	**Silent infection (n = 30)**	**Minor infection (n = 27)**	**Severe infection (n = 34)**	***P*-value**
**mDC/PBMC, %**	0.53 ± 0.13	AP	1.09 ± 0.34^b^	0.81 ± 0.48^b,c^	0.53 ± 0.24^c,d^	<0.0001^a^
		CP	0.89 ± 0.43^b,e^	ND	0.70 ± 0.30^e^	0.0014^a^
**pDC/PBMC, %**	0.24 ± 0.11	AP	0.42 ± 0.21^b^	0.39 ± 0.23^b^	0.22 ± 0.14^c,d^	<0.0001^a^
		CP	0.37 ± 0.22^b^	ND	0.38 ± 0.12^b,e^	0.0048^a^
**IFN-γ**^**+**^**CD4/CD3, %**	23.09 ± 6.03	AP	17.41 ± 7.75^b^	16.13 ± 5.60^b,d^	23.94 ± 6.72^c,d^	<0.0001^a^
		CP	23.38 ± 6.81^e^	ND	24.50 ± 8.73	0.0001^a^
**IFN-γ + CD8/CD3, %**	14.30 ± 5.65	AP	15.88 ± 8.17	12.53 ± 6.33	18.30 ± 8.23	0.0251
		CP	23.30 ± 8.94^b,e^	ND	18.40 ± 8.66^d^	0.0003^a^
**IL-17**^**+**^**CD4/CD3, %**	2.06 ± 0.94	AP	1.74 ± 0.96	1.93 ± 0.89^d^	2.84 ± 1.19^b,c,d^	0.0004^a^
		CP	3.37 ± 1.47^b,e^	ND	3.04 ± 1.36^b^	0.0002^a^
**IL-17**^**+**^**CD8/CD3, %**	0.22 ± 0.17	AP	0.54 ± 0.33^b^	0.40 ± 0.22^b,d^	0.25 ± 0.18^c^	<0.0001^a^
		CP	0.81 ± 0.34^b^	ND	0.74 ± 0.69^b^	<0.0001^a^

DC, dendritic cell; AP, acute phase; PBMC, peripheral blood mononuclear cell; CP, convalescent phase; ND, not determined.

^a^significant differences in the four groups by one-way ANOVA.

^b^significant difference from the healthy control group.

^c^significant difference from the silent infection group.

^d^significant difference from the minor infection group.

^e^significant difference from the AP within a group.

In the AP, patients with severe infections had the highest median level of IL-17^+^CD4^+^ cells (*p* < 0.05 for all comparisons; Figure 1 and Table 4). In contrast, the silent and minor infection groups had higher levels of IL-17^+^CD8^+^ cells than the control group at baseline, and the severe infection group had lower levels of these cells than the silent infection group. Finally, the silent and severe infection groups had higher levels of IL-17^+^CD4^+^ and IL-17^+^CD8^+^ cells at the CP relative to their levels at baseline and relative to those in the control and minor infection groups (Table 4).

### Effect of HAdV-55 infection on cytokine expression

Figure 3 shows the levels of 6 cytokines in the different groups during the AP and CP. In the AP, the minor and severe infection groups had significantly higher levels of IFN-γ, IL-10, and IFN-α2 than the healthy control and silent infection groups. The severe infection group had a higher level of IL-4 than all other groups, and the silent and minor infection groups had a higher level of IL-4 relative to the control group. The severe infection group had a significantly higher level of IL-15 than the control group. The silent and severe infection groups had higher levels of MCP-3 relative to the control group, but there was no significant difference between the control and minor infection groups.

The severe infection group had significantly lower levels of IFN-γ, IL-4, IL-10, IL-15, and IFN-α2 during the CP than during the AP. The three groups (control, silent infection, severe infection) had no significant differences in the levels of any of the tested cytokines during the CP. Additional file 1: Table S3 summarizes the data for all of the cytokines analyzed during the AP and CP.

## Discussion

This purpose of this cross-sectional study was to elucidate the clinical characteristics of patients with HAdV-55 infections as a first step toward the identification of biomarkers that can be used to predict disease severity. Our results indicated that patients with silent infections, minor infections, severe infections, and healthy controls had significant differences in the proportions of neutrophils, lymphocytes, monocytes, and PLT counts during the AP of HAdV-55 infection. Patients with more severe infections had more neutrophils, fewer lymphocytes, and significantly lower PLT counts. In addition, the silent and minor infection groups had significantly higher mDC and pDC levels. Patients with severe disease had increased levels of IL-17^+^CD4^+^ cells, decreased levels of IL-17^+^CD8^+^ cells, and higher levels of IFN-γ, IL-4, and IL-10. In addition to HAdV-55 outbreaks, there have been other reports of outbreaks of HAdV types in which pneumonia was observed in immunocompetent adults [20-22]. In particular, Cao et al. [22] compared the epidemiological and clinical characteristics of Chinese patients infected with different HAdV types and found that patients with HAdV-55 infections were older and had more severe pneumonia than patients infected by other HAdV types. However, these other studies focused on the epidemiology and clinical features of the infection rather than peripheral blood cell profiles of infected patients. The present study is the first to analyze the peripheral blood cell profiles of patients with varying severities of HAdV-55 infections during the AP and CP and to identify potential biomarkers that may ultimately be used to predict disease severity.

HAdV infection leads to neutrophil accumulation and then monocyte infiltration of the lungs [11,12]. In the present study, we observed increased serum levels of neutrophils and monocytes in the severe infection group at the AP. In the CP, the proportions of neutrophils and monocytes significantly decreased to the levels of the healthy control group. Generally, there is a transient increase of neutrophils following an infection. This is consistent with our observation of elevated levels of neutrophils and monocytes during the AP, and decreased levels during the CP. These changes are similar to those reported for infection by Type A H1N1 [23]. Further studies of target organs, such as the lungs, will be needed to determine if increased levels of these cells correspond with pulmonary infiltration.

In the AP, patients with silent infections had higher PLT counts than healthy controls, but the minor and severe infection groups had PLT counts similar to the healthy controls. However, in the CP, the severe infection group had a significantly elevated PLT count relative to the healthy controls. PLTs can produce thrombocidins, antimicrobial peptides that are produced in response to infectious agents [24]. Thus, it is possible that the elevated PLT count in the silent infection group during the early stage of the infection may have contributed to their absence of symptoms.

DCs are critical for the activation of the immune response to AdV infection [25,26]. AdV infection causes pDCs to produce large amounts of type I interferon, which has pleiotropic anti-viral effects in the early phase of infection. The mDCs present the virus antigen to adaptive cells, which secrete interleukins (mainly IL-12) that activate other immune cells. We observed increased levels of mDCs and pDCs in the silent and minor infection groups during the AP, but not in patients with severe infections. However, in the CP, the levels of mDCs and pDCs increased in the severe infection group to levels comparable to those of the silent infection group at that time.

Patients with severe HAdV-55 infections had increased levels of Th17 cells. A previous study of severe respiratory syncytial virus (RSV) infection indicated that neutralization of IL-17 significantly reduced inflammation and viral counts, possibly due to increased CD8 cytotoxic T cell activation [27]. Another study reported that reductions in Th17 cells were associated with T cell activation in patients with H1N1 influenza A infection [28]. Studies of the role of IL-17 in the pathogenesis of this virus may be useful for the design of treatments.

Patients with different severities of HAdV-55 infection expressed different proinflammatory cytokines. For example, the severe infection group had higher levels of IL-10 than the silent infection group during the AP. Thus, it is possible that more regulatory T cells were induced or activated in patients with severe infections, possibly due to an inhibition of the anti-viral response. Previous studies in children with HAdV infections, including HAdV-3 and HAdV-7 [29-31], reported no differences in the levels of IL-10 in controls and infected patients, suggesting that this response may be unique to HAdV-55 infection. Interestingly, we only observed increased IFN-γ levels in patients with severe infections during the AP. This is consistent with other *in vivo* studies of AdV infections [11,32] and with the transient increase of IFN-γ in H1N1 influenza infections [33].

Pathogens are routinely collected from oropharyngeal swabs of patients with respiratory tract infections. Clinical diagnosis is based on symptoms, signs, and epidemiological data and laboratory diagnosis is based on etiological and immunological data. Not all cases are suitable for etiological examination because sample time is crucial. If a pathogen is detected and a clinical diagnosis is indicated, there can be a definitive diagnosis. Similarly, specific immunological data can generally be used to make a definitive diagnosis. However, for certain diseases in which there is no known production of specific antibodies or available diagnostic test, it is necessary to combine clinical signs with laboratory data for diagnosis. A positive pathogen test result alone cannot be used to determine disease severity. The low number of pathogens in our silent infection group demonstrated that viral copy number was low or that there was rapid virus elimination due to a strong immune response. In the present study, we only observed HAdV-55 DNA in 41% of patients with severe infections and 30% of patients with minor infections, so this parameter cannot be used to indicate infection severity or disease progression. Analysis of IgG and IgM-specific AdV antibodies was more reliable in the identification of patients with AdV infections. Furthermore, analysis of the HAdV55-specific IgM IF score indicated that patients with severe and silent HAdV-55 infections had significantly higher scores than those with minor infections. It is possible that patients with silent infections had strong immune responses (high IF scores) and this prevented clinical symptoms; patients with minor infections had weak immune responses (low IF scores) and some clinical symptoms; and patients with severe infections had strong immune responses, but a higher viral load that made their disease more severe. This explanation is consistent with our observation that more patients with severe disease had evidence of HAdV-55 DNA (Table 1) and became more immunocompromised (Additional file 1: Table S3) than the other groups. Further studies are needed to confirm this explanation.

This study is limited in that only peripheral blood was sampled for analysis, and there were no samples from infected organs, such as the lungs. Thus, it is unclear if the altered cell counts that we observed were due to changes in the total number of cells or due to migration of cells to target organ(s). Furthermore, if alterations in PBLs are to be used to predict disease progression, it is necessary to have more detailed information about whether the PBL parameters changed before, during, or after manifestations of severe disease. Another limitation is that although there were several HAdV-55 outbreaks during this time period in China, we only examined one outbreak. Thus, these results need to be confirmed by studies of other outbreaks of HAdV-55. Finally, our study was limited to infection by one HAdV-55 serotype, so the results should not be generalized to other serotypes.

## Conclusion

This is the first study to analyze the peripheral blood cell profiles of patients with HAdV-55 infections. Patients with silent, minor, and severe infections had significant differences in IgM IF scores, blood cell counts, lymphocyte subsets, and cytokine levels. These results provide a foundation for use of the immunological profiles of patients with HAdV-55 infections for estimation of prognosis.

## Abbreviations

HAdV-55: Human adenovirus serotype 55; FRI: Febrile respiratory illness; TNF-a: Tumor necrosis factor alpha; IL: Interleukin; IL-1b: Interleukin-1 beta; IFN: Interferon; MCP-3: Monocyte chemotactic protein-3; MIP1: Macrophage inflammatory protein 1; AP: Acute phase; CP: Convalescent phase; IgM: Immunoglobulin M; IgG: Immunoglobulin G; CPE: Cytopathic effects; IF: Immunofluorescence staining; FITC: Fluorescein isothiocyanate; PE: Phycoerythrin; APC: Allophycocyanine; Percp: Peridinin-chlorophyll-protein complex; NK: Nature killer cells; pDC: Plasmacytoid dendritic cells; mDC: Myeloid dendritic cells; Th17: T help cell 17; PLT: Platelet; WBC: White blood cell.

## Competing interests

The authors declare that they have no competing interests.

## Authors’ contributions

WC, JL and MZ conceived the study, participated in its design and coordination, and helped to draft the manuscript. WC carried out the immunoassays and participated in the statistical analysis. WN and WX carried out the molecular genetic studies and immunoassays, participated in the design of the study, drafted the manuscript, and participated in the sequence alignment. YX, BT, and PZ participated in the clinical data acquisition. EQ and YZ participated in the data analysis. XZ, WL and ZZ performed the statistical analysis. All authors read and approved the final manuscript.

## Pre-publication history

The pre-publication history for this paper can be accessed here:

http://www.biomedcentral.com/1471-2334/14/147/prepub

## Supplementary Material

Additional file 1: Table S1 Changes of IgG titers from the acute phase to the convalescent phase in the patients of silent infection and severe infection groups. **Table S2.** Lymphocyte subsets of the different groups of patients in the AP and CP. **Table S3.** Cytokine levels of the different groups of patients in the AP and CP. Click here for file
