# Supplementary material for: Cross-sectional study of the relationship of peripheral blood cell profiles with severity of infection by adenovirus type 55
Source: BMC Infect Dis. 2014 Mar 19;14:147. doi: 10.1186/1471-2334-14-147 (PMC4000060; doi:10.1186/1471-2334-14-147)
Supplement: Additional file 1: Table S1 — Changes of IgG titers from the acute phase to the convalescent phase in the patients of silent infection and severe infection groups. Table S2. Lymphocyte subsets of the different groups of patients in the AP and CP. Table S3. Cytokine levels of the different groups of patients in the AP and CP. [file 1471-2334-14-147-S1.doc]

**Additional file 1: Table S1**. Changes of IgG titers from the acute phase to the convalescent phase in the patients of silent infection and severe infection groups.

.

|  | **Silent infection (N=30)** | | |  | **Severe infection (N=34)** | | |
| --- | --- | --- | --- | --- | --- | --- | --- |
| **Subject**  **No.** | **Acute phase**  **IgG (U/mL)** | **Convalescent phase**  **IgG (U/mL)** | **Fold**  **increase** |  | **Acute phase**  **IgG (U/mL)** | **Convalescent phase**  **IgG (U/mL)** | **Fold**  **increase** |
| 1 | 19 | 75 | 4.0 |  | 237 | 1247 | 5.3 |
| 2 | 49 | 41 | 0.8 |  | 105 | 420 | 4.0 |
| 3 | 75 | 62 | 0.8 |  | 21 | 90 | 4.3 |
| 4 | 175 | 175 | 1.0 |  | 77 | 439 | 5.7 |
| 5 | 47 | 50 | 1.1 |  | 41 | 196 | 4.8 |
| 6 | 60 | 52 | 0.9 |  | 79 | 157 | 2.0 |
| 7 | 64 | 59 | 0.9 |  | 48 | 251 | 5.2 |
| 8 | 93 | 101 | 1.1 |  | 76 | 126 | 1.7 |
| 9 | 155 | 136 | 0.9 |  | 83 | 96 | 1.2 |
| 10 | 194 | 126 | 0.6 |  | 58 | 74 | 1.3 |
| 11 | 91 | 93 | 1.0 |  | 53 | 215 | 4.1 |
| 12 | 65 | 59 | 0.9 |  | 37 | 217 | 5.9 |
| 13 | 128 | 112 | 0.9 |  | 156 | 188 | 1.2 |
| 14 | 145 | 650 | 4.5 |  | 71 | 94 | 1.3 |
| 15 | 140 | 136 | 1.0 |  | 103 | 117 | 1.1 |
| 16 | 23 | 27 | 1.2 |  | 56 | 345 | 6.2 |
| 17 | 36 | 177 | 4.9 |  | 113 | 137 | 1.2 |
| 18 | 125 | 130 | 1.0 |  | 154 | 132 | 0.9 |
| 19 | 26 | 23 | 0.9 |  | 64 | 89 | 1.4 |
| 20 | 235 | 171 | 0.7 |  | 137 | 187 | 1.4 |
| 21 | 54 | 43 | 0.8 |  | 57 | 430 | 7.6 |
| 22 | 64 | 66 | 1.0 |  | 31 | 151 | 4.9 |
| 23 | 142 | 137 | 1.0 |  | 46 | 180 | 3.9 |
| 24 | 126 | 77 | 0.6 |  | 204 | 142 | 0.7 |
| 25 | 22 | 117 | 5.3 |  | 53 | 72 | 1.4 |
| 26 | 41 | 107 | 2.6 |  | 101 | 96 | 1.0 |
| 27 | 114 | 120 | 1.1 |  | 45 | 214 | 4.8 |
| 28 | 23 | 106 | 4.6 |  | 25 | 162 | 6.4 |
| 29 | 67 | 59 | 0.9 |  | 31 | 183 | 6.0 |
| 30 | 29 | 115 | 4.0 |  | 61 | 99 | 1.6 |
| 31 |  |  |  |  | 60 | 94 | 1.6 |
| 32 |  |  |  |  | 40 | 166 | 4.1 |
| 33 |  |  |  |  | 51 | 75 | 1.5 |
| 34 |  |  |  |  | 36 | 149 | 4.1 |

**Additional file 1: Table S2.** Lymphocyte subsets of the different groups of patients in the AP and CP.

| **Cell type** | **Healthy controls  (n=30)** | **Stage** | **Silent infection  (n=30)** | **Minor infection (n=27)** | **Severe infection (n=34)** | ***P*-value** |
| --- | --- | --- | --- | --- | --- | --- |
| **Lymphocyte cells/mm^3^** | 1,717.93 ± 763.78 | AP | 2,352.37 ± 604.88^b^ | 1,692.26 ± 556.83^c,d^ | 1,479.77 ± 468.78^c^ | <0.0001^a^ |
|  |  | CP | 2,162.40 ± 530.98^b,e^ | ND | 2,266.63 ± 523.13^b,d,e^ | 0.0002^a^ |
| **CD3**  **%** | 65.43 ± 6.91 | AP | 68.40 ± 6.02 | 69.07 ± 7.16 | 68.20 ± 8.38 | 0.2256 |
|  |  | CP | 67.07 ± 5.97 | ND | 64.53 ± 8.76 | 0.1001 |
| **CD3**  **cells/mm^3^** | 1,104.53 ± 465.24 | AP | 1,606.43 ± 438.94^b^ | 1,185.22 ± 468.58^c^ | 1,014.60 ± 354.88^c^ | <0.0001^a^ |
|  |  | CP | 1,458.37 ± 411.15^b^ | ND | 1,465.40 ± 390.36^b^ | 0.0016^a^ |
| **CD4**  **%** | 33.10 ± 4.87 | AP | 32.47 ± 5.77 | 33.52 ± 6.07 | 33.27 ± 7.04 | 0.9210 |
|  |  | CP | 34.60 ± 6.11 | ND | 30.90 ± 5.89 | 0.0938 |
| **CD4**  **cells/mm^3^** | 524.80 ± 170.74 | AP | 760.53 ± 254.77^b^ | 570.04 ± 224.04^c,d^ | 493.93 ± 181.39^c^ | <0.0001^a^ |
|  |  | CP | 758.77 ± 253.29^b^ | ND | 681.57 ± 166.00^b^ | <0.0001^a^ |
| **CD8**  **%** | 26.13 ± 6.22 | AP | 30.67 ± 5.77 | 30.00 ± 6.40 | 31.07 ± 6.68 | 0.0108 |
|  |  | CP | 28.77 ± 6.01 | ND | 27.97 ± 6.05 | 0.1197 |
| **CD8**  **cells/mm^3^** | 417.73 ± 177.75 | AP | 734.47 ± 264.51^b^ | 517.41 ± 244.95^c^ | 463.73 ± 198.35^c^ | <0.0001^a^ |
|  |  | CP | 626.77 ± 203.63^b^ | ND | 622.43 ± 203.56^b^ | 0.0003^a^ |
| **B cell**  **%** | 12.67 ± 3.54 | AP | 8.97 ± 2.97^b^ | 11.41 ± 3.78 | 12.87 ± 4.31^c^ | 0.0002^a^ |
|  |  | CP | 12.10 ± 4.13^e^ | ND | 10.33 ± 3.48^e^ | 0.0964 |
| **B cell**  **cells/mm^3^** | 238.87 ± 198.44 | AP | 206.33 ± 81.05 | 194.37 ± 111.49 | 189.63 ± 96.37 | 0.4658 |
|  |  | CP | 268.62 ± 113.65^e^ | ND | 234.20 ± 83.61^e^ | 0.2374 |
| **NK**  **%** | 20.07 ± 6.89 | AP | 19.27 ± 6.41 | 16.48 ± 6.23 | 15.67 ± 7.87 | 0.0427 |
|  |  | CP | 16.48 ± 6.03^e^ | ND | 21.20 ± 9.34^e^ | 0.0242 |
| **NK**  **cells/mm^3^** | 382.37 ± 320.18 | AP | 458.63 ± 201.87 | 261.59 ± 102.61^c^ | 230.27 ± 149.64^b,c^ | 0.0001^a^ |
|  |  | CP | 344.17 ± 128.63^e^ | ND | 491.20 ± 256.44^e^ | 0.0021^a^ |
| **CD4/CD8** | 1.34 ± 0.36 | AP | 1.11 ± 0.37 | 1.19 ± 0.39 | 1.14 ± 0.44 | 0.1213 |
|  |  | CP | 1.28 ± 0.43 | ND | 1.18 ± 0.40 | 0.3375 |

Data are presented as mean ± standard deviation (SD). NK, natural killer cell; ND, not determined

^a^ significant differences in the four groups by one-way ANOVA.

^b^ significant difference from the healthy control group.

^c^ significant difference from the silent infection group.

^d^ significant difference from the minor infection group.

^e^ significant difference from the AP within a group

.

**Additional file 1: Table S3.** Cytokine levels of the different groups of patients in the AP and CP.

|  | **Healthy control** | **Stage** | **Silent infection** | **Minor infection** | **Severe infection** | **P-value** |
| --- | --- | --- | --- | --- | --- | --- |
| **Fractalkine (pg/ml)** | 5.57 (<4.74, 18.08) | AP | 14.25 (<4.74, 145.00) | 19.21 (<4.74, 97.75)^b^ | 16.67 (5.23, 119.00)^b^ | <0.0001^a^ |
|  |  | CP | 10.89 (<4.74, 26.01) | ND | 14.25 (<4.74, 45.89)^b^ | 0.0038^a^ |
| **IFN-α2 (pg/ml)** | 3.42 (<3.41, 10.09) | AP | 5.64 (<3.41, 10.58) | 22.94 (11.35, 41.20)^bc^ | 19.03 (7.89, 172.00)^bc^ | <0.0001^a^ |
|  |  | CP | 5.28 (<3.41, 13.28) | ND | 7.89 (<3.41, 20.17)^be^ | 0.0009^a^ |
| **IFN-γ (pg/ml)** | <1.00 (<1.00, 1.76) | AP | 1.28 (<1.00, 5.61)^b^ | 9.45 (1.59, 47.86)^bc^ | 5.97 (1.76, 21.92)^bc^ | <0.0001^a^ |
|  |  | CP | 1.36 (<1.00, 2.60) | ND | 1.28 (<1.00, 13.36)^e^ | 0.0427^a^ |
| **IL-1β (pg/ml)** | all <1.10 | AP | all <1.10 | <1.10 (<1.10, 3.23) | <1.10 (<1.10, 4.57) | 0.6166 |
|  |  | CP | all <1.10 | ND | all <1.10 | NA |
| **IL-2 (pg/ml)** | all <1.56 | AP | all <1.56 | <1.56 (<1.56, 2.17) | <1.56 (<1.56, 4.57) | 0.3992 |
|  |  | CP | all <1.56 | ND | all <1.56 | NA |
| **IL-4 (pg/ml)** | <1.73 (1.38, 8.53) | AP | 5.17 (<1.73, 13.25)^b^ | 7.09 (<1.73, 18.17)^b^ | 14.61 (5.91, 24.07)^bcd^ | <0.0001^a^ |
|  |  | CP | 1.89 (<1.73, 11.10)^e^ | ND | 2.15 (<1.73, 13.12)^e^ | 0.2663 |
| **IL-5 (pg/ml)** | all <1.53 | AP | all <1.53 | all <1.53 | all <1.53 | NA |
|  |  | CP | all <1.53 | ND | all <1.53 | NA |
| **IL-6 (pg/ml)** | all <0.28 | AP | <0.28 (<0.28, 15.27) | <0.28 (<0.28, 5.13) | <0.28 (<0.28, 31.66)^b^ | 0.0108^a^ |
|  |  | CP | <0.28 (<0.28, 12.33) | ND | <0.28 (<0.28, 0.44)^e^ | 0.6297 |
| **IL-8 (pg/ml)** | 2.32 (1.14, 4.43) | AP | 3.34 (0.53, 35.21) | 5.33 (2.25, 13.23)^b^ | 15.13 (3.25, 41.25)^bc^ | <0.0001^a^ |
|  |  | CP | 4.98 (1.30, 20.81)^b^ | ND | 7.84 (3.47, 41.55)^b^ | <0.0001^a^ |
| **IL-10 (pg/ml)** | ^a^ll <2.05 | AP | <2.05 (<2.05, 4.34) | 5.79 (<2.05, 18.46)^bc^ | 5.20 (<2.05, 127.00)^bc^ | <0.0001a |
|  |  | CP | <2.05 (<2.05, 2.61) | ND | <2.05 (<2.05, 107.00)^e^ | 0.0908 |
| **IL-15 (pg/ml)** | all <1.90 | AP | all <1.90 | <1.90 (<1.90, 3.84) | <1.90 (<1.90, 9.32)^b^ | 0.0009^a^ |
|  |  | CP | all <1.90 | ND | <1.90 (<1.90, 7.03)^e^ | 1.0000 |
| **IL-17 (pg/ml)** | all <0.33 | AP | 0.44 (<0.33, 40.44)^b^ | 0.38 (<0.33, 4.51)^b^ | 0.66 (<0.33, 3.43)^b^ | <0.0001^a^ |
|  |  | CP | 0.36 (<0.33, 22.92)^b^ | ND | 0.58 (<0.33, 2.63)^b^ | <0.0001^a^ |
| **MCP-3 (pg/ml)** | all <3.69 | AP | <3.69 (<3.69, 5.70)^b^ | <3.69 (3.51, 6.34) | 4.26 (<3.69, 17.64)^b^ | 0.0043^a^ |
|  |  | CP | all <3.69 ^e^ | ND | <3.69 (<3.69, 30.62) | 0.0080^a^ |
| **MIP1-A (pg/ml)** | <0.40 (<0.40, 4.32) | AP | 8.30 (<0.40, 15.59)^b^ | 5.85 (<0.40, 14.82)^b^ | 10.51 (<0.40, 56.68)^b^ | 0.0001^a^ |
|  |  | CP | 6.85 (<0.40, 11.74)^be^ | ND | 12.47 (<0.40, 17.72)^be^ | <0.0001^a^ |
| **MIP1-B (pg/ml)** | 18.67 (9.41, 27.48) | AP | 41.89 (22.01, 91.92)^b^ | 25.24 (12.17, 36.61)^c^ | 35.81 (14.02, 68.36)^bd^ | <0.0001^a^ |
|  |  | CP | 31.53 (16.43, 71.00)^be^ | ND | 41.23 (9.80, 73.54)^b^ | <0.0001^a^ |
| **TNF-α (pg/ml)** | 3.93 (1.31, 8.38) | AP | 6.14 (1.68, 16.84) | 9.19 (4.35, 15.00)^b^ | 10.43 (4.29, 17.15)^bc^ | <0.0001^a^ |
|  |  | CP | 5.26 (1.35, 14.78)^e^ | ND | 7.24 (3.16, 13.79)^be^ | 0.0085^a^ |
| **IL-12 (pg/ml)** | all <1.26 | AP | <1.26 (<1.26, 5.34)^b^ | <1.26 (<1.26, 5.43)^b^ | <1.26 (<1.26, 5.34) | 0.0238^a^ |
|  |  | CP | <1.26 (<1.26, 2.79) | ND | <1.26 (<1.26, 20.03)^b^ | 0.0227^a^ |

Data are presented as median and full range.

^a^ significant differences in the four groups by Kruskal-Wallis test.

^b^ significant difference from the healthy control group.

^c^ significant difference from the silent infection group.

^d^ significant difference from the minor infection group.

^e^ significant difference from the AP within a group.
